# Bis(5-hy­droxy­isophthalato-κ*O* ^1^)bis­[4-(pyridine-3-carboxamido-κ*N* ^3^)pyridinium]copper(II) tetra­hydrate

**DOI:** 10.1107/S1600536813030675

**Published:** 2013-11-16

**Authors:** Megan E. O’Donovan, Robert L. LaDuca

**Affiliations:** a Lyman Briggs College, Department of Chemistry, Michigan State University, East Lansing, MI 48825, USA

## Abstract

In the title compound, [Cu(C_11_H_10_N_3_O)_2_(C_8_H_4_O_5_)_2_]·4H_2_O, the Cu^II^ ion, located on a crystallographic inversion center, is coordinated in a square-planar environment by two *trans*-O atoms belonging to two monodentate 5-hy­droxy­isophthalate (hip) dianions and two *trans* nicotinamide pyridyl N-donor atoms from monodentate protonated pendant *N*-(pyridin-4-yl)nicotinamide (4-pnaH) ligands. The protonated 4-pyridyl­amine groups engage in N—H^+^⋯O^−^ hydrogen-bond donation to unligated hip O atoms to construct supra­molecular chain motifs parallel to [100]. Water mol­ecules of crystallization, situated between the chains, engage in O—H⋯O hydrogen bonding to form supra­molecular layers and the overall three-dimensional network structure.

## Related literature

For the preparation of 4-pyridyl­nicotinamide, see: Gardner *et al.* (1954 ▶). For the preparation of other di­carboxyl­ate coordin­ation polymers containing 4-pyridyl­nicotinamide, see: Kumar (2009 ▶); Wilson *et al.* (2013 ▶)
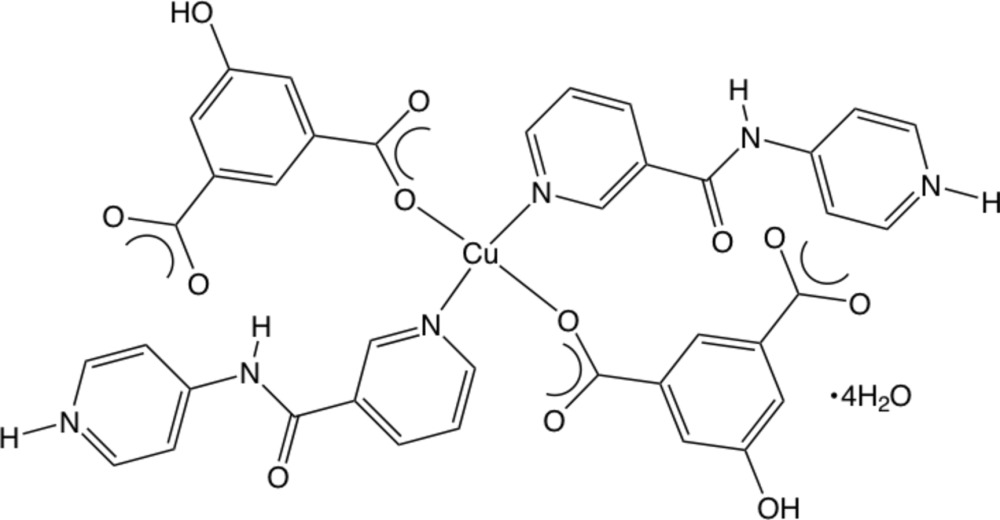


## Experimental

### 

#### Crystal data

[Cu(C_11_H_10_N_3_O)_2_(C_8_H_4_O_5_)_2_]·4H_2_O
*M* *_r_* = 896.27
Monoclinic, 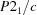

*a* = 16.402 (2) Å
*b* = 7.7699 (10) Å
*c* = 16.403 (2) Å
β = 115.466 (1)°
*V* = 1887.3 (4) Å^3^
*Z* = 2
Mo *K*α radiation
μ = 0.67 mm^−1^
*T* = 173 K
0.50 × 0.20 × 0.18 mm

#### Data collection

Bruker APEXII CCD diffractometer
Absorption correction: multi-scan (*SADABS*; Bruker, 2012 ▶) *T*_min_ = 0.686, *T*_max_ = 0.745
15084 measured reflections
3483 independent reflections
3122 reflections with *I* > 2σ(*I*)
*R*_int_ = 0.025

#### Refinement

*R*[*F* ^2^ > 2σ(*F* ^2^)] = 0.027
*wR*(*F* ^2^) = 0.076
*S* = 1.07
3483 reflections
288 parameters
H atoms treated by a mixture of independent and constrained refinement
Δρ_max_ = 0.26 e Å^−3^
Δρ_min_ = −0.42 e Å^−3^

### 

Data collection: *APEX2* (Bruker, 2006 ▶); cell refinement: *SAINT* (Bruker, 2012 ▶); data reduction: *SAINT*; program(s) used to solve structure: *OLEX2* (Dolomanov *et al.*, 2009 ▶); program(s) used to refine structure: *SHELXL97* (Sheldrick, 2008 ▶); molecular graphics: *CrystalMaker* (Palmer, 2007 ▶); software used to prepare material for publication: *OLEX2*.

## Supplementary Material

Crystal structure: contains datablock(s) I, p21c. DOI: 10.1107/S1600536813030675/hg5361sup1.cif

Structure factors: contains datablock(s) I. DOI: 10.1107/S1600536813030675/hg5361Isup2.hkl

Additional supplementary materials:  crystallographic information; 3D view; checkCIF report

## Figures and Tables

**Table 1:** Hydrogen-bond geometry (Å, °)

*D*—H⋯*A*	*D*—H	H⋯*A*	*D*⋯*A*	*D*—H⋯*A*
O1*W*—H1*WA*⋯O4^i^	0.87	1.85	2.7164 (18)	170
O1*W*—H1*WB*⋯O3^ii^	0.87	1.92	2.775 (2)	169
O2*W*—H2*WA*⋯O2	0.87	1.97	2.814 (2)	163
O2*W*—H2*WB*⋯O2^iii^	0.87	1.94	2.8049 (18)	177
O5—H5⋯O1*W*	0.84	1.81	2.6384 (18)	168
N2—H2⋯O2*W* ^iv^	0.88	2.00	2.823 (2)	156
N3—H3⋯O3^v^	0.91 (3)	1.69 (3)	2.582 (2)	166 (2)

Symmetry codes: (i) 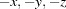; (ii) 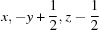; (iii) 
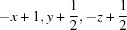
; (iv) 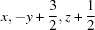; (v) 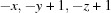.

**Fig. 1.**
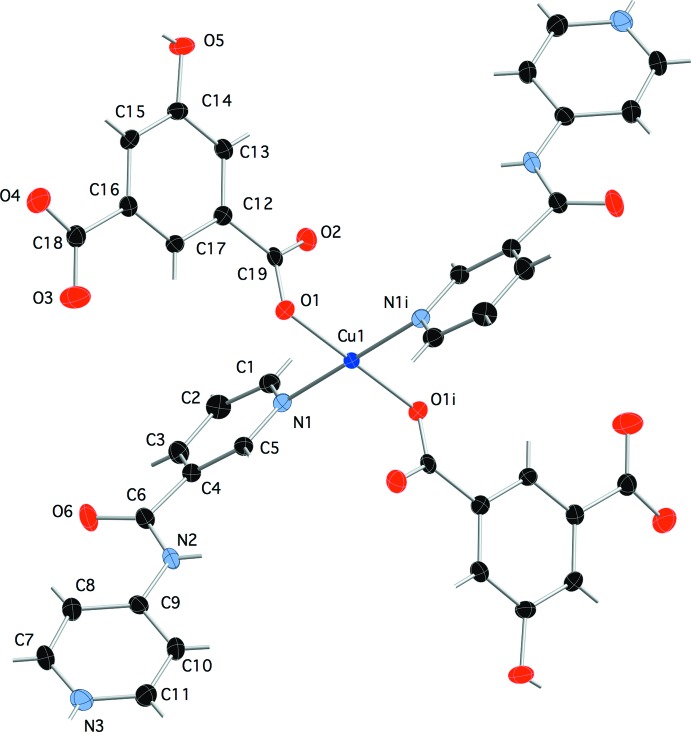
A complete molecule of the title compound, showing 50% probability ellipsoids, and atom numbering scheme. Hydrogen atom positions are shown as grey sticks. Color codes: dark blue Cu, red O, light blue N, black C. Symmetry code: (i) -x + 1, -y + 1, -z + 1.

**Fig. 2.**
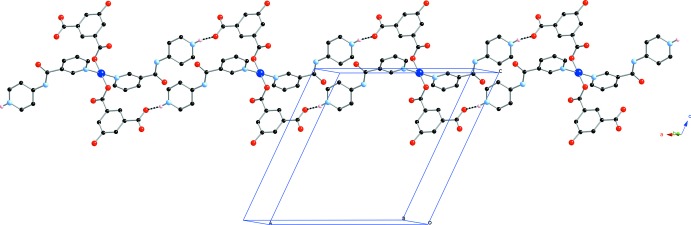
A single supramolecular chain in the title compound. N—H+···O- hydrogen bonding is shown as dashed lines.

**Fig. 3.**
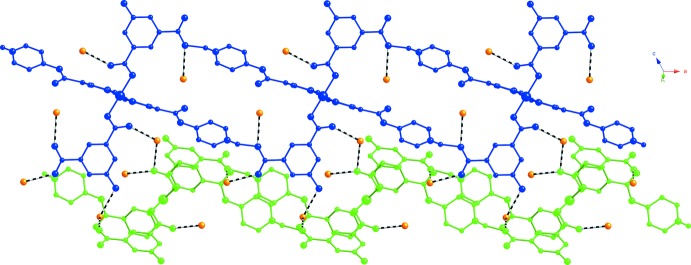
Aggregation of supramolecular chains in the title compound, mediated by water molecules of crystallization (orange spheres). O—H···O hydrogen bonding is shown as dashed lines.

**Fig. 4.**
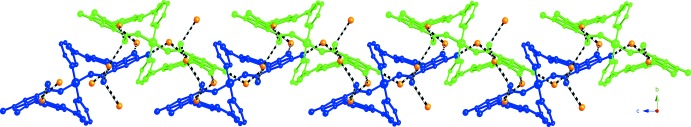
Layer of supramolecular chains in the title compound. O—H···O hydrogen bonding is shown as dashed lines.

**Fig. 5.**
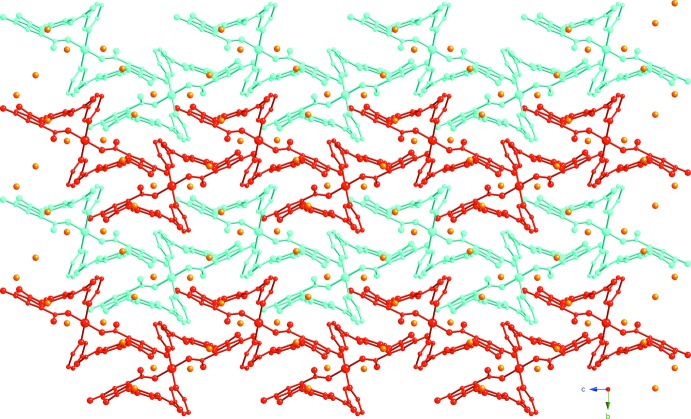
Stacking of supramolecular layers within the title compound.

## supplementary crystallographic information

### 1. Comment

In comparison to divalent metal coordination polymers containing rigid rod dipyridine ligands such as 4,4'-bipyridine, related phases containing the kinked and hydrogen-bonding capable dipodal ligand 4-pyridylnicotinamide (4-pna) are less widely reported (Kumar, 2009; Wilson *et al.*, 2013). The title compound was obtained in attempt at preparing a coordination polymer, as purple crystals through the hydrothermal reaction of copper nitrate, 5-hydroxyisophthalic acid (H2hip), and 4-pna in the presence of aqueous base.

The asymmetric unit of the title compound, which exists as a simple coordination complex [Cu(hip)_2_(4-pnaH)_2_]·4H_2_O, contains a divalent copper atom on a crystallographic inversion center, a 4-pnaH ligand protonated at its unligated 4-pyridylamine terminus, a doubly deprotonated hip ligand, and two water molecules of crystallization. The copper atom is square planar coordinated (Fig. 1) by two *trans* O atoms belonging to two monodentate 5-hydroxyisophthalate (hip) dianions and two *trans* nicotinamide pyridyl N-donor atoms from 4-pnaH ligands.

Neighboring [Cu(hip)_2_(4-pnaH)_2_] coordination complexes are connected into supramolecular chains parallel to [1 0 0] by charge-separated N—H^+^···O^-^ hydrogen bonding between the protonated termini of the 4-pnaH ligands and unligated hip oxygen atoms (Fig. 2). These chains aggregate into undulating supramolecular layers (Fig. 3) by means of O—H···O hydrogen bonding mediated by the water molecules of crystallization. The main interchain aggregation mechanism involves O—H···O hydrogen bonding from water molecules (O2W) to unbound hip oxygen atoms (O2) belonging to the ligated monodentate carboxylate groups (Fig. 4). These water molecules also accept N—H···O hydrogen bonding from the central amide functional group of the 4-pnaH ligands. The aggregation of the supramolecular layers into the full three-dimensional crystal structure of the title compound is accomplished by O—H···O hydrogen bonding patterns. The hydroxyl group of the hip ligands in one layer donates hydrogen bonds to the water molecules of crystallization (O1W), which in turn serve as hydrogen bonding donors to unligated hip oxygen atoms in a neighboring layer (Fig. 5).

### 2. Experimental

Copper(II) nitrate hydrate and 5-hydroxyisophthalic acid were obtained commercially. 4-Pyridylnicotinamide (4-pna) was prepared *via* a published procedure (Gardner *et al.*, 1954). A mixture of copper nitrate hydrate (65 mg, 0.28 mmol), 5-hydroxyisophthalic acid (51 mg, 0.28 mmol), 4-pna (55 mg, 0.28 mmol) and 10.0 g water (550 mmol), along with 0.5 mL of 1.0 M NaOH solution was placed into a 23 ml Teflon-lined Parr acid digestion bomb, which was then heated under autogenous pressure at 373 K for 48 h. Purple blocks of the title compound were obtained.

### 3. Refinement

All H atoms bound to C atoms were placed in calculated positions, with C—H = 0.95 Å, and refined in riding mode with *U*_iso_ = 1.2*U*_eq_(C). The H atom within the amide group of the 4-pna ligand was found in a difference Fourier map, restrained with N—H = 0.9 Å and refined with *U*_iso_ = 1.2*U*_eq_(N).

### Crystal data

**Table:**

[Cu(C_11_H_10_N_3_O)_2_(C_8_H_4_O_5_)_2_]·4H_2_O	*F*(000) = 926
*M**_r_* = 896.27	*D*_x_ = 1.577 Mg m^−^^3^
Monoclinic, *P*2_1_/*c*	Mo *K*α radiation, λ = 0.71073 Å
*a* = 16.402 (2) Å	Cell parameters from 9855 reflections
*b* = 7.7699 (10) Å	θ = 2.5–25.4°
*c* = 16.403 (2) Å	µ = 0.67 mm^−^^1^
β = 115.466 (1)°	*T* = 173 K
*V* = 1887.3 (4) Å^3^	Block, purple
*Z* = 2	0.50 × 0.20 × 0.18 mm

### Data collection

**Table:**

Bruker APEXII CCD diffractometer	3483 independent reflections
Radiation source: fine-focus sealed tube	3122 reflections with *I* > 2σ(*I*)
Graphite monochromator	*R*_int_ = 0.025
φ and ω scans	θ_max_ = 25.4°, θ_min_ = 2.5°
Absorption correction: multi-scan (*SADABS*; Bruker, 2012)	*h* = −19→19
*T*_min_ = 0.686, *T*_max_ = 0.745	*k* = −9→9
15084 measured reflections	*l* = −19→19

### Refinement

**Table:**

Refinement on *F*^2^	Primary atom site location: iterative
Least-squares matrix: full	Secondary atom site location: difference Fourier map
*R*[*F*^2^ > 2σ(*F*^2^)] = 0.027	Hydrogen site location: inferred from neighbouring sites
*wR*(*F*^2^) = 0.076	H atoms treated by a mixture of independent and constrained refinement
*S* = 1.07	*w* = 1/[σ^2^(*F*_o_^2^) + (0.0359*P*)^2^ + 1.2489*P*] where *P* = (*F*_o_^2^ + 2*F*_c_^2^)/3
3483 reflections	(Δ/σ)_max_ < 0.001
288 parameters	Δρ_max_ = 0.26 e Å^−^^3^
0 restraints	Δρ_min_ = −0.42 e Å^−^^3^

### Special details

**Table:**

Experimental. SADABS-2012/1 (Bruker,2012) was used for absorption correction. wR2(int) was 0.0479 before and 0.0373 after correction. The Ratio of minimum to maximum transmission is 0.9208. The λ/2 correction factor is 0.0015.
Geometry. All e.s.d.'s (except the e.s.d. in the dihedral angle between two l.s. planes) are estimated using the full covariance matrix. The cell e.s.d.'s are taken into account individually in the estimation of e.s.d.'s in distances, angles and torsion angles; correlations between e.s.d.'s in cell parameters are only used when they are defined by crystal symmetry. An approximate (isotropic) treatment of cell e.s.d.'s is used for estimating e.s.d.'s involving l.s. planes.
Refinement. Refinement of *F*^2^ against ALL reflections. The weighted *R*-factor *wR* and goodness of fit *S* are based on *F*^2^, conventional *R*-factors *R* are based on *F*, with *F* set to zero for negative *F*^2^. The threshold expression of *F*^2^ > σ(*F*^2^) is used only for calculating *R*-factors(gt) *etc*. and is not relevant to the choice of reflections for refinement. *R*-factors based on *F*^2^ are statistically about twice as large as those based on *F*, and *R*- factors based on ALL data will be even larger.

### Fractional atomic coordinates and isotropic or equivalent isotropic displacement parameters (Å^2^)

**Table:**

	*x*	*y*	*z*	*U*_iso_*/*U*_eq_	
Cu1	0.5000	0.5000	0.5000	0.01401 (10)	
O1	0.39722 (8)	0.39435 (15)	0.40222 (8)	0.0194 (3)	
O1W	0.14145 (9)	−0.00460 (18)	−0.10490 (9)	0.0275 (3)	
H1WA	0.0933	−0.0485	−0.1035	0.041*	
H1WB	0.1260	0.0626	−0.1516	0.041*	
O2	0.46616 (8)	0.48157 (16)	0.31902 (9)	0.0228 (3)	
O2W	0.40905 (9)	0.7846 (2)	0.21591 (10)	0.0321 (3)	
H2WA	0.4346	0.6894	0.2425	0.048*	
H2WB	0.4483	0.8481	0.2072	0.048*	
O3	0.07175 (9)	0.2777 (2)	0.25064 (10)	0.0376 (4)	
O4	0.01886 (9)	0.13283 (19)	0.12102 (9)	0.0304 (3)	
O5	0.27672 (8)	0.14834 (18)	0.02882 (8)	0.0255 (3)	
H5	0.2295	0.1022	−0.0096	0.038*	
O6	0.15526 (9)	0.9500 (2)	0.45393 (9)	0.0310 (3)	
N1	0.42685 (9)	0.71203 (18)	0.47758 (9)	0.0162 (3)	
N2	0.23450 (10)	0.7950 (2)	0.58285 (10)	0.0209 (3)	
H2	0.2868	0.7446	0.6140	0.025*	
N3	0.05618 (10)	0.7640 (2)	0.69925 (11)	0.0243 (3)	
H3	0.0179 (18)	0.754 (3)	0.7256 (17)	0.049 (7)*	
C1	0.43854 (12)	0.8397 (2)	0.42900 (11)	0.0195 (4)	
H1	0.4862	0.8312	0.4109	0.023*	
C2	0.38399 (13)	0.9829 (2)	0.40438 (13)	0.0241 (4)	
H2A	0.3935	1.0713	0.3694	0.029*	
C3	0.31522 (13)	0.9966 (2)	0.43120 (13)	0.0227 (4)	
H3A	0.2769	1.0947	0.4152	0.027*	
C4	0.30281 (11)	0.8649 (2)	0.48184 (11)	0.0184 (4)	
C5	0.35943 (11)	0.7231 (2)	0.50298 (11)	0.0174 (3)	
H5A	0.3503	0.6312	0.5363	0.021*	
C6	0.22328 (12)	0.8746 (2)	0.50426 (12)	0.0212 (4)	
C7	0.02556 (12)	0.8174 (3)	0.61421 (13)	0.0272 (4)	
H7	−0.0363	0.8480	0.5824	0.033*	
C8	0.08076 (12)	0.8293 (3)	0.57121 (13)	0.0255 (4)	
H8	0.0576	0.8666	0.5102	0.031*	
C9	0.17144 (12)	0.7858 (2)	0.61853 (12)	0.0196 (4)	
C10	0.20149 (12)	0.7265 (3)	0.70722 (12)	0.0246 (4)	
H10	0.2627	0.6932	0.7407	0.029*	
C11	0.14239 (13)	0.7166 (3)	0.74539 (13)	0.0272 (4)	
H11	0.1627	0.6755	0.8055	0.033*	
C12	0.32288 (11)	0.3368 (2)	0.24693 (11)	0.0165 (3)	
C13	0.24036 (11)	0.3152 (2)	0.25072 (11)	0.0167 (3)	
H13	0.2332	0.3524	0.3024	0.020*	
C14	0.16865 (11)	0.2397 (2)	0.17922 (11)	0.0174 (4)	
C15	0.17939 (11)	0.1822 (2)	0.10391 (11)	0.0183 (4)	
H15	0.1306	0.1283	0.0554	0.022*	
C16	0.26192 (12)	0.2041 (2)	0.10003 (11)	0.0183 (4)	
C17	0.33334 (11)	0.2836 (2)	0.17084 (12)	0.0187 (4)	
H17	0.3891	0.3015	0.1674	0.022*	
C18	0.07858 (12)	0.2138 (2)	0.18280 (12)	0.0218 (4)	
C19	0.40097 (11)	0.4108 (2)	0.32675 (11)	0.0167 (4)	

### Atomic displacement parameters (Å^2^)

**Table:**

	*U*^11^	*U*^22^	*U*^33^	*U*^12^	*U*^13^	*U*^23^
Cu1	0.01290 (16)	0.01534 (16)	0.01324 (16)	0.00149 (11)	0.00510 (12)	−0.00120 (10)
O1	0.0185 (6)	0.0214 (6)	0.0162 (6)	−0.0007 (5)	0.0053 (5)	−0.0027 (5)
O1W	0.0196 (7)	0.0403 (9)	0.0210 (7)	−0.0074 (6)	0.0074 (6)	−0.0019 (6)
O2	0.0155 (6)	0.0255 (7)	0.0267 (7)	−0.0033 (5)	0.0084 (5)	−0.0035 (5)
O2W	0.0195 (7)	0.0429 (9)	0.0321 (8)	−0.0053 (6)	0.0096 (6)	0.0088 (7)
O3	0.0245 (7)	0.0638 (10)	0.0321 (8)	−0.0103 (7)	0.0193 (7)	−0.0163 (7)
O4	0.0189 (7)	0.0450 (9)	0.0263 (7)	−0.0103 (6)	0.0088 (6)	−0.0057 (6)
O5	0.0225 (7)	0.0359 (8)	0.0209 (7)	−0.0062 (6)	0.0119 (6)	−0.0104 (6)
O6	0.0217 (7)	0.0413 (8)	0.0309 (8)	0.0147 (6)	0.0121 (6)	0.0113 (6)
N1	0.0156 (7)	0.0166 (7)	0.0149 (7)	−0.0006 (6)	0.0051 (6)	−0.0022 (5)
N2	0.0148 (7)	0.0278 (8)	0.0207 (8)	0.0068 (6)	0.0081 (6)	0.0018 (6)
N3	0.0181 (8)	0.0325 (9)	0.0260 (8)	−0.0007 (7)	0.0130 (7)	−0.0050 (7)
C1	0.0183 (9)	0.0224 (9)	0.0188 (9)	−0.0021 (7)	0.0090 (7)	−0.0018 (7)
C2	0.0288 (10)	0.0189 (9)	0.0270 (10)	−0.0012 (8)	0.0142 (8)	0.0041 (7)
C3	0.0239 (10)	0.0178 (9)	0.0246 (10)	0.0050 (7)	0.0086 (8)	0.0019 (7)
C4	0.0170 (8)	0.0206 (9)	0.0167 (8)	0.0014 (7)	0.0062 (7)	−0.0017 (7)
C5	0.0172 (8)	0.0190 (8)	0.0164 (8)	0.0001 (7)	0.0076 (7)	0.0002 (7)
C6	0.0180 (9)	0.0222 (9)	0.0231 (9)	0.0029 (7)	0.0086 (8)	−0.0013 (7)
C7	0.0149 (9)	0.0362 (11)	0.0288 (10)	0.0041 (8)	0.0079 (8)	−0.0011 (8)
C8	0.0188 (9)	0.0342 (11)	0.0224 (9)	0.0045 (8)	0.0078 (8)	0.0017 (8)
C9	0.0171 (9)	0.0205 (9)	0.0218 (9)	0.0008 (7)	0.0091 (7)	−0.0057 (7)
C10	0.0153 (9)	0.0350 (11)	0.0218 (9)	0.0032 (8)	0.0064 (8)	0.0005 (8)
C11	0.0211 (9)	0.0399 (12)	0.0207 (9)	0.0012 (8)	0.0089 (8)	−0.0003 (8)
C12	0.0171 (8)	0.0146 (8)	0.0169 (8)	0.0008 (7)	0.0064 (7)	0.0014 (7)
C13	0.0178 (8)	0.0181 (8)	0.0143 (8)	0.0020 (7)	0.0069 (7)	0.0009 (7)
C14	0.0161 (8)	0.0180 (8)	0.0183 (8)	0.0003 (7)	0.0077 (7)	0.0034 (7)
C15	0.0165 (8)	0.0205 (9)	0.0151 (8)	−0.0018 (7)	0.0040 (7)	−0.0008 (7)
C16	0.0214 (9)	0.0192 (9)	0.0158 (8)	0.0008 (7)	0.0093 (7)	−0.0004 (7)
C17	0.0154 (8)	0.0203 (9)	0.0216 (9)	0.0004 (7)	0.0091 (7)	0.0009 (7)
C18	0.0178 (9)	0.0282 (10)	0.0199 (9)	−0.0003 (8)	0.0085 (8)	0.0031 (7)
C19	0.0146 (8)	0.0130 (8)	0.0207 (9)	0.0036 (7)	0.0058 (7)	−0.0017 (7)

### Geometric parameters (Å, º)

**Table:**

Cu1—O1	1.9399 (12)	C2—C3	1.380 (3)
Cu1—O1^i^	1.9399 (12)	C3—H3A	0.9500
Cu1—N1^i^	1.9772 (14)	C3—C4	1.387 (3)
Cu1—N1	1.9773 (14)	C4—C5	1.386 (2)
O1—C19	1.272 (2)	C4—C6	1.502 (2)
O1W—H1WA	0.8701	C5—H5A	0.9500
O1W—H1WB	0.8701	C7—H7	0.9500
O2—C19	1.256 (2)	C7—C8	1.369 (3)
O2W—H2WA	0.8698	C8—H8	0.9500
O2W—H2WB	0.8700	C8—C9	1.392 (2)
O3—C18	1.267 (2)	C9—C10	1.398 (3)
O4—C18	1.236 (2)	C10—H10	0.9500
O5—H5	0.8400	C10—C11	1.364 (3)
O5—C16	1.362 (2)	C11—H11	0.9500
O6—C6	1.216 (2)	C12—C13	1.392 (2)
N1—C1	1.337 (2)	C12—C17	1.394 (2)
N1—C5	1.342 (2)	C12—C19	1.498 (2)
N2—H2	0.8800	C13—H13	0.9500
N2—C6	1.369 (2)	C13—C14	1.385 (2)
N2—C9	1.392 (2)	C14—C15	1.394 (2)
N3—H3	0.91 (3)	C14—C18	1.517 (2)
N3—C7	1.330 (3)	C15—H15	0.9500
N3—C11	1.337 (2)	C15—C16	1.393 (2)
C1—H1	0.9500	C16—C17	1.390 (2)
C1—C2	1.375 (3)	C17—H17	0.9500
C2—H2A	0.9500		
			
O1—Cu1—O1^i^	180.0	N3—C7—C8	121.77 (17)
O1^i^—Cu1—N1^i^	87.52 (5)	C8—C7—H7	119.1
O1—Cu1—N1^i^	92.48 (5)	C7—C8—H8	120.6
O1^i^—Cu1—N1	92.48 (5)	C7—C8—C9	118.76 (18)
O1—Cu1—N1	87.52 (5)	C9—C8—H8	120.6
N1^i^—Cu1—N1	180.00 (7)	N2—C9—C10	117.46 (15)
C19—O1—Cu1	111.94 (11)	C8—C9—N2	124.21 (16)
H1WA—O1W—H1WB	109.5	C8—C9—C10	118.32 (16)
H2WA—O2W—H2WB	109.5	C9—C10—H10	120.2
C16—O5—H5	109.5	C11—C10—C9	119.61 (17)
C1—N1—Cu1	119.87 (11)	C11—C10—H10	120.2
C1—N1—C5	119.10 (15)	N3—C11—C10	120.81 (18)
C5—N1—Cu1	120.71 (11)	N3—C11—H11	119.6
C6—N2—H2	116.7	C10—C11—H11	119.6
C6—N2—C9	126.57 (15)	C13—C12—C17	120.07 (16)
C9—N2—H2	116.7	C13—C12—C19	119.32 (15)
C7—N3—H3	120.0 (16)	C17—C12—C19	120.56 (15)
C7—N3—C11	120.68 (17)	C12—C13—H13	120.0
C11—N3—H3	119.3 (16)	C14—C13—C12	120.09 (15)
N1—C1—H1	118.9	C14—C13—H13	120.0
N1—C1—C2	122.24 (16)	C13—C14—C15	120.09 (16)
C2—C1—H1	118.9	C13—C14—C18	120.67 (15)
C1—C2—H2A	120.5	C15—C14—C18	119.21 (16)
C1—C2—C3	119.10 (17)	C14—C15—H15	120.1
C3—C2—H2A	120.5	C16—C15—C14	119.81 (16)
C2—C3—H3A	120.5	C16—C15—H15	120.1
C2—C3—C4	119.02 (16)	O5—C16—C15	122.39 (16)
C4—C3—H3A	120.5	O5—C16—C17	117.44 (15)
C3—C4—C6	118.46 (16)	C17—C16—C15	120.16 (15)
C5—C4—C3	118.77 (16)	C12—C17—H17	120.1
C5—C4—C6	122.53 (16)	C16—C17—C12	119.74 (16)
N1—C5—C4	121.75 (16)	C16—C17—H17	120.1
N1—C5—H5A	119.1	O3—C18—C14	115.95 (16)
C4—C5—H5A	119.1	O4—C18—O3	125.54 (16)
O6—C6—N2	124.73 (16)	O4—C18—C14	118.50 (16)
O6—C6—C4	119.88 (16)	O1—C19—C12	115.53 (15)
N2—C6—C4	115.40 (15)	O2—C19—O1	122.79 (15)
N3—C7—H7	119.1	O2—C19—C12	121.66 (15)
			
Cu1—O1—C19—O2	2.7 (2)	C7—N3—C11—C10	1.9 (3)
Cu1—O1—C19—C12	−178.48 (11)	C7—C8—C9—N2	−179.01 (18)
Cu1—N1—C1—C2	173.90 (14)	C7—C8—C9—C10	2.0 (3)
Cu1—N1—C5—C4	−174.92 (13)	C8—C9—C10—C11	−1.5 (3)
O1—Cu1—N1—C1	−99.24 (13)	C9—N2—C6—O6	0.2 (3)
O1^i^—Cu1—N1—C1	80.76 (13)	C9—N2—C6—C4	179.90 (16)
O1—Cu1—N1—C5	74.23 (13)	C9—C10—C11—N3	−0.4 (3)
O1^i^—Cu1—N1—C5	−105.77 (13)	C11—N3—C7—C8	−1.3 (3)
O5—C16—C17—C12	−177.43 (15)	C12—C13—C14—C15	1.1 (3)
N1—Cu1—O1—C19	91.90 (11)	C12—C13—C14—C18	179.40 (15)
N1^i^—Cu1—O1—C19	−88.10 (11)	C13—C12—C17—C16	−2.0 (3)
N1—C1—C2—C3	0.5 (3)	C13—C12—C19—O1	23.5 (2)
N2—C9—C10—C11	179.44 (18)	C13—C12—C19—O2	−157.68 (16)
N3—C7—C8—C9	−0.6 (3)	C13—C14—C15—C16	−1.3 (3)
C1—N1—C5—C4	−1.4 (2)	C13—C14—C18—O3	5.6 (3)
C1—C2—C3—C4	−0.3 (3)	C13—C14—C18—O4	−173.92 (17)
C2—C3—C4—C5	−0.7 (3)	C14—C15—C16—O5	179.04 (16)
C2—C3—C4—C6	−175.17 (16)	C14—C15—C16—C17	−0.2 (3)
C3—C4—C5—N1	1.6 (3)	C15—C14—C18—O3	−176.14 (17)
C3—C4—C6—O6	28.3 (3)	C15—C14—C18—O4	4.4 (3)
C3—C4—C6—N2	−151.50 (17)	C15—C16—C17—C12	1.9 (3)
C5—N1—C1—C2	0.3 (3)	C17—C12—C13—C14	0.5 (2)
C5—C4—C6—O6	−146.00 (19)	C17—C12—C19—O1	−154.05 (15)
C5—C4—C6—N2	34.2 (2)	C17—C12—C19—O2	24.8 (2)
C6—N2—C9—C8	12.5 (3)	C18—C14—C15—C16	−179.57 (16)
C6—N2—C9—C10	−168.55 (18)	C19—C12—C13—C14	−177.03 (15)
C6—C4—C5—N1	175.84 (15)	C19—C12—C17—C16	175.49 (15)

Symmetry code: (i) −*x*+1, −*y*+1, −*z*+1.

### Hydrogen-bond geometry (Å, º)

**Table:**

*D*—H···*A*	*D*—H	H···*A*	*D*···*A*	*D*—H···*A*
O1*W*—H1*WA*···O4^ii^	0.87	1.85	2.7164 (18)	170
O1*W*—H1*WB*···O3^iii^	0.87	1.92	2.775 (2)	169
O2*W*—H2*WA*···O2	0.87	1.97	2.814 (2)	163
O2*W*—H2*WB*···O2^iv^	0.87	1.94	2.8049 (18)	177
O5—H5···O1*W*	0.84	1.81	2.6384 (18)	168
N2—H2···O2*W*^v^	0.88	2.00	2.823 (2)	156
N3—H3···O3^vi^	0.91 (3)	1.69 (3)	2.582 (2)	166 (2)

Symmetry codes: (ii) −*x*, −*y*, −*z*; (iii) *x*, −*y*+1/2, *z*−1/2; (iv) −*x*+1, *y*+1/2, −*z*+1/2; (v) *x*, −*y*+3/2, *z*+1/2; (vi) −*x*, −*y*+1, −*z*+1.
